# Three Novel Xanthones from *Garcinia* *paucinervis* and Their Anti-TMV Activity

**DOI:** 10.3390/molecules18089663

**Published:** 2013-08-13

**Authors:** Yu-Ping Wu, Wei Zhao, Zhen-Yuan Xia, Guang-Hui Kong, Xiu-Ping Lu, Qiu-Fen Hu, Xue-Mei Gao

**Affiliations:** 1 Yunnan Academy of Tobacco Agricultural Sciences, Yuxi 653100, China; E-Mails: ypwumm@163.com (Y.-P.W.); zyxia@yntsti.com (Z.-Y.X.); 13908776036@163.com (G.-H.K.); xplu1970@163.com (X.-P.L.); huqiufena@aliyun.com (Q.-F.H.); 2 Key Laboratory of Tobacco Chemistry of Yunnan Province, Yunnan Academy of Tobacco Science, Kunming 650106, China; E-Mail: zhaoweizhaorong@126.com; 3 Key Laboratory of Chemistry in Ethnic Medicinal Resources, State Ethnic Affairs Commission and Ministry of Education, Yunnan University of Nationalities, Kunming 650031, China

**Keywords:** *Garciniapaucinervis*, xanthones, anti-tobacco mosaic virus

## Abstract

Phytochemical investigations of the leaves of *Garcinia* *paucinervis* resulted in the isolation of three new xanthones **1**–**3** and five known analogues **4**–**8**. Structural elucidations of **1**–**3** were performed by spectral methods such as 1D and 2D (HMQC, HMBC, and ROESY) NMR spectroscopy, in addition to high resolution mass spectrometry. Compounds **1**–**3** showed anti-TMV activities, with inhibition rates above 20%, especially for **1**, which had a lower IC_50_ value of 21.4 µM.

## 1. Introduction

**Figure 1:**
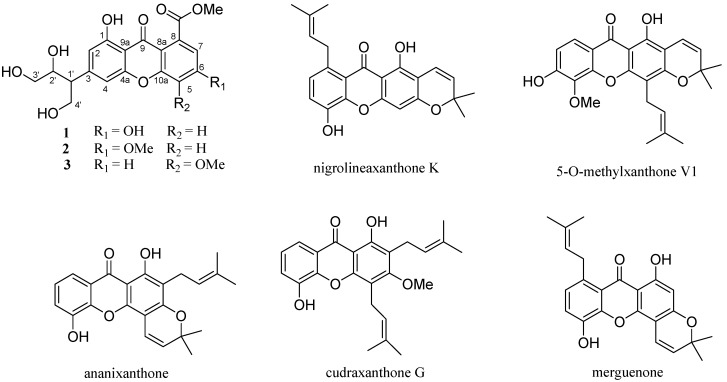
The structures of compounds **1**–**8**.

The genus *Garcinia* (Guttiferae family) is known to be a rich source of polyisoprenylated benzophenones and xanthones, some of which have shown various biological activities such as antibacterial [1], antifungal [2], anti-inflammatory [3], antioxidant [4], apoptosis-inducing [5,6], and cytotoxic effects [7]. *Garcinia paucinervis* is a valuable species distributed in the Yunnan and Guangxi provinces of China [8]. The present studies on chemical constituents of the acetone extract of the dried leaves of *G*. *paucinervis* afforded three new xanthones **1**–**3** (Figure 1), and five known analogues, namely nigrolineaxanthone K (**4**) [9], 5-*O*-methylxanthone V1 (**5**) [10], ananixanthone (**6**) [11], cudraxanthone G (**7**) [12], and merguenone (**8**) [13] (Figure 1). In this paper, we describe the isolation, structure elucidation, and anti-tobacco mosaic virus (anti-TMV) activities of these compounds. 

## 2. Results and Discussion

**Figure 2:**
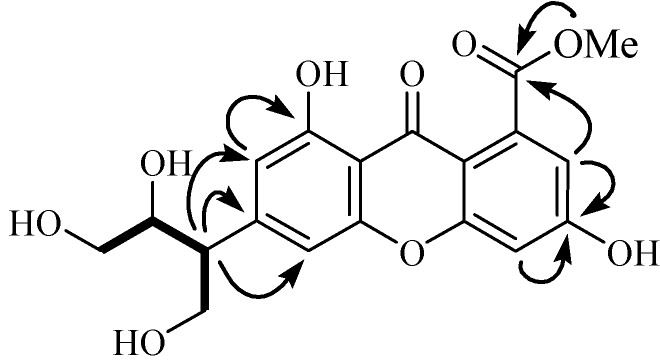
The key HMBC and COSY correlations of compound **1**.

Compound **1** was obtained as a yellow amorphous powder. Its molecular formula was determined to be C_19_H_18_O_9_ on the basis of positive HR-ESI-MS (calcd. for [*M*+Na]^+^ *m*/*z* 413.0842; found, 413.0849) with 11 degrees of unsaturation. The UV absorptions at 310, 246, and 210 nm showed an extended chromophore and a substituted benzene ring, suggesting a xanthone skeleton. Its IR spectral data showed the presence of hydroxy groups (3,415 cm^−1^) and phenyl groups (1,658, 1,584 and 1,548 cm^−1^). The ^1^H-NMR spectrum (Table 1) displayed four aromatic methines [*δ_H_* 6.75 (1H, s, H-2), 7.00 (1H, s, H-4), 6.89 (1H, s, H-5), 6.80 (1H, s, H-7)], one methoxy group (*δ_H_* 4.04), two oxygenated methylenes [*δ_H_* 4.63 (2H, m, H-3') and 4.13 and 4.19 (2H, m, H-4')], and two methines, including one oxygenated *δ_H_* 5.14 (1H, m, H-2') and one non-oxygenated [*δ_H_* 4.41 (1H, m, H-1')] one, and two hydroxy protons [*δ_H_* 13.07 (1H, s, HO-1), *δ_H_* 12.83 (1H, s, HO-6)]. Further analysis of its ^13^C-NMR (DEPT) data (Table 1) revealed the presence of one ketone and one ester carbonyls, twelve aromatic carbons (four of which were protonated) indicative of two substituted phenyl rings, one methoxy group, two methylenes, and two methines. By careful analysis of the above data, we concluded that **1** was a xanthone analogue. One spin coupling system from C-1' to C-4' was deduced from correlations between H-4'/H-1'/H-2'/H-3' observed in the ^1^H, ^1^H-COSY spectrum, and the -H(CH_2_OH)CHOHCH_2_OH group was connected at C-3 according to the HMBC correlations from H-1' to C-2, C-3, and C-4. This fragment is very rare in xanthones, and it should be a degradation product of an isopentene group. The two hydroxy groups were located at C-1 and C-6 positions, as deduced from the HMBC correlations (Figure 2) of H-2 with C-1, and of H-5 and H-7 with C-6. HMBC correlations from OMe (*δ_H_* 4.04, 3H, s) and H-7 (*δ_H_* 6.80, 1H, s) to a carbonyl (*δ_C_* 169.4, s) suggested that the methyl ester was connected at C-8. From above information, the gross structure of **1** was established as paucinervin E (Figure 1).

**Table 1:** ^1^H- (500 MHz) and ^13^C-NMR (125 MHz) data for **1**–**3** (*δ* in ppm and *J* in Hz, data recorded in C_5_D_5_N).

No.	1		2		3	
	*δ*_C_ (m)	*δ*_H_ (m, *J*, Hz)	*δ*_C_ (m)	*δ*_H_ (m, *J*, Hz)	*δ*_C_ (m)	*δ*_H_ (m, *J*, Hz)
1	162.7 s		161.8 s		161.4 s	
2	108.5 d	6.75 s	108.8 d	6.75 s	110.0 d	6.94 d (1.8)
3	149.1 s		149.0 s		145.2 s	
4	104.1 d	7.00 s	104.8 d	7.00 s	108.8 d	7.07 d (1.8)
5	103.0 d	6.89 s	102.3 d	6.93 s	156.0 s	
6	165.1 s		167.1 s		121.5 d	7.70 d (8.8)
7	113.8 d	6.80 s	112.7 d	6.84 s	125.2 d	7.39 d (8.8)
8	136.8 s		137.1 s		126.5 s	
9	181.9 s		181.8 s		181.5 s	
4a	155.6 s		155.0 s		157.0 s	
8a	109.2 s		109.7 s		119.4 s	
9a	107.4 s		107.7 s		107.8 s	
10a	158.4 s		157.2 s		146.8 s	
1′	42.1 d	4.41 m	43.0 d	4.44 m	41.8 d	4.42 m
2′	72.3 d	5.14 m	72.0 d	5.29 m	72.0 d	5.13 m
3′	66.0 t	4.63 m	66.0 t	4.74 m	66.2 t	4.64, 4.71 m
4′	61.8 t	4.13, 4.19 m	62.0 t	4.20 m	61.8 t	4.17, 4.22 m
5′	169.4 s		169.7 s		169.0 s	
5-OMe					55.9 q	3.79 s
6-OMe			56.2 q	3.80 s		
5′-OMe	52.4 q	4.04 s	52.5 q	4.11 s	52.5 q	4.02 s
Ar-OH-1		13.07 s		13.79 s		13.22 s
Ar-OH-6		12.83 s				

Compound **2**, a yellow amorphous powder, was assigned the molecular formula C_20_H_20_O_9_, based on the HR-ESI-MS which revealed the [*M*+Na]^+^ peak at *m/z* 427.1101 (calcd. for C_20_H_20_NaO_9_^+^. 427.1005). Its UV and IR absorption bands were very similar to those of **1**. Analysis of the ^13^C-NMR, DEPT, and HSQC spectra revealed that **2** also contained one ketone and one ester carbonyls, twelve aromatic carbons (four of which were protonated) indicative of two substituted phenyl rings, two methoxy groups, two methylenes, and two methines. By careful comparison, we found that the ^13^C-NMR spectral data of **2** were almost identical to those of **1**, expect for the presence of one more methoxy group in **2**. The key HMBC correlation of H-(MeO) (*δ_H_* 3.80, 3H, s) with C-6 (*δ_C_* 167.1, s) indicated a methoxy group at C-6 in **2**, instead of a hydroxy group at C-6 in **1**. Finally, compound **2** was identified as paucinervin F (Figure 1).

Compound **3**, obtained as a yellow amorphous powder, gave the molecular formula C_20_H_20_O_9_, as revealed by its HR-ESI-MS at *m/z* 427.1001 [*M*+Na]^+^ (calcd. for C_20_H_20_NaO_9_^+^. 427.1005). Its UV, IR, ^1^H, and ^13^C were very similar to **2**: one ketone and one ester carbonyls, twelve aromatic carbons (four of which were protonated) indicative of two substituted phenyl rings, two methoxy groups, two methylenes, and two methines. The only difference between **3** and **2** is that the methoxy group was at C-5 in **3** instead of at C-6 in **2**, which was futher confirmed by the key HMBC correlation of H-(MeO) (*δ_H_* 3.79, 3H, s) with C-6 (*δ_C_* 156.0, s). Thus, compound **3** was finally identified as paucinervin G (Figure 1).

Since the C-C bonds can rotate randomly, it is very difficult to determine the relative configurations of C-1' and C-2' through only spectroscopic analysis. We tried to determine the relative configuration of **1** by CD spectra and X-ray crystallography, but unfortunately, we failed to obtain the desired results, and the relative configurations of C-1' and C-2' in compounds **1**–**3** thus remain unassigned.

By comparison with NMR data in literatures, five known analogues, namely nigrolineaxanthone K (**4**) [9], 5-*O*-methylxanthone V1 (**5**) [10], ananixanthone (**6**) [11], cudraxanthone G (**7**) [12], and merguenone (**8**) [13], were identified.

Compounds **1**–**8** were tested for their anti-TMV activity using the half-leaf method [14,15]. Ningnanmycin, a commercial biochemical pesticide used against virus diseases on tomato, pepper, melons, tobacco, and many other crops with high efficiency, was used as a positive control. The antiviral inhibition rates of compounds **1**–**8** at the concentration of 20 μM are listed in Table 2. Compounds **1**–**3** showed anti-TMV activities with inhibition rate above 20%. Compound **1** is more active with lower IC_50_ value of 21.4 µM perhaps because of the presence of hydroxy groups. The interactions through hydrogen bonding with other moieties would be the main factor.

**Table 2:** Anti-TMV Activity of **1**–**7** on *Garcinia* *paucinervis* Leaf ^a^.

No.	% Inhibition at 20 *µ*M	IC_50_ (*µ*M)
1	43.2 ± 2.3	21.4 ± 2.3
2	28.7 ± 3.0	42.8 ± 3.0
3	24.8 ± 2.2	53.6 ± 2.2
4	9.22 ± 2.8	≥200
5	18.9 ± 2.6	82.4 ± 2.6
6	17.8 ± 2.3	68.9 ± 2.3
7	16.1 ± 3.0	52.8 ± 3.0
8	13.1 ± 3.0	≥200
Ningnamycin	30.5 ± 2.4	36.9 ± 2.4

^a^ All results are expressed as mean ± SD; *n* = 3.

## 3. Experimental

### 3.1. General

Ultraviolet absorption spectra were recorded using a Perkin-Elmer Lambda L14 spectrometer. A Perkin Elmer Spectrum 100 FT-IR spectrometer was used for scanning IR spectroscopy with KBr pellets. The ^1^D and ^2^D NMR spectra were recorded on a Bruker AV-400 spectrometer with TMS as the internal standard. Chemical shifts (*δ*) were expressed in ppm with reference to the solvent signals. HRMS were obtained using a nanoLC-MS/MS system, with a nanoAcquity ultra-performance liquid chromatography (UPLC) module and a quadrupole time-of-flight (Q-TOF) spectrometer equipped with a nanoelectrospray ion source (Waters, Milford, MA, USA) and connected to a lock-mass apparatus to perform a real-time calibration correction. Column chromatography was performed with silica gel (200–300 mesh, Qingdao Marine Chemical, Inc., Qingdao, China), Sephadex LH-20 (Pharmacia, Sweden), and reversed-phase C_18_ silica gel (250 meshes, Merck, Germany). Precoated TLC sheets of silica gel 60 GF_254_ were used. An Agilent 1100 series equipped with an Alltima C_18_ column (4.6 × 250 mm) was used for HPLC analysis, and semipreparative and preparative Alltima C18 columns or Zorbax SB-C18 columns (9.4 × 250 mm and 22 × 250 mm) were used in sample preparation. Spots were visualized by heating silica gel plates sprayed with 10% H_2_SO_4_ in EtOH.

### 3.2. Plant Material

The leaves of *Garcinia* *paucinervis* were collected in October 2010 from the district of XiShuangBanNa Prefecture, Yunnan Province, China. The plant was identified by Pan-Yu Ren. A voucher specimen has been deposited at the Key Laboratory of Ethnic Medicine Resource Chemistry (Yunnan University of Nationalities), State Ethnic Affairs Commission & Ministry of Education.

### 3.3. Extraction and Isolation

An acetone extract prepared from the leaves of *Garcinia* *paucinervis* (5.5 kg) was decolorized by MCI GEL(polystyrene polymer based packing material) and chromatographed on a silica gel column eluting with hexane/acetone (1:0, 4:1, 2:1, 1:1, and 0:1) to afford five fractions A–E. Further separation of fraction B (42 g) on silica gel, eluted with petroleum ether–acetone (9:1–1:2), yielded fractions B1–B7. Fraction B2 (6.28 g) was subjected to silica gel column chromatography using petroleum ether-acetone followed by semipreparative HPLC (78% MeOH–H_2_O, flow rate 12 mL/min) to give **3** (1.8 mg), **5** (42.7 mg), and **7** (33.3 mg). Fraction B3 (5.72 g), upon separation on silica gel using petroleum ether-acetone and semipreparative HPLC (70% MeOH–H_2_O, flow rate 12 mL/min), afforded **1** (1.6 mg), **4** (11.8 mg), **6** (14.6 mg). Fraction B4 (11.6 g) was subjected to silica gel column chromatography using petroleum ether–acetone and semipreparative HPLC (65% MeOH–H_2_O, flow rate 12 mL/min) to yield **2** (1.2 mg), **8** (3.6 mg).

*Paucinervin* *E* (**1**): Yellow amorphous powder, UV (MeOH) λ_max_ (log *ε*): 210 (4.30), 246 (3.62), 310 (3.94) nm; IR (KBr) v_max_: 3,415, 3,076, 2,923, 2,865, 1,725, 1,658, 1,584, 1,548, 1,460, 1,372, 1,128, 1,076, 878, 729 cm^–1^; ^1^H- and ^13^C-NMR: see Table 1; ESIMS *m/z* (positive ion mode) 413 [M+Na]^+^; HRESIMS (positive ion mode) *m/z* 413.0842 [M+Na]^+^ (calcd. C_19_H_18_NaO_9_ for 413.0849). 

*Paucinervin* *F* (**2**): Yellow amorphous powder, UV (MeOH) *λ*_max_ (log *ε*) 210 (4.38), 248 (3.57), 312 (4.01) nm; IR (KBr) *ν*_max_ 3,418, 3,080, 2,919, 2,872, 1,729, 1,655, 1,593, 1,543, 1,462, 1,375, 1,125, 1,074, 882, 725 cm^–1^; ESIMS *m/z* (positive ion mode) 427 [M+Na]^+^; HRESIMS (positive ion mode) *m/z* 427.1011 [M+Na]^+^ (calcd. C_20_H_20_NaO_9_ for 427.1005). 

*Paucinervin* *G* (**3**): Yellow amorphous powder, UV (MeOH) *λ*_max_ (log *ε*) 210 (4.25), 263 (3.76), 310 (3.92) nm; IR (KBr) *ν*_max_ 3,422, 3,085, 2,914, 2,867, 1,725, 1,652, 1,598, 1,540, 1,457, 1,379, 1,226, 1,138, 1,076, 886, 748 cm^–1^; ESIMS *m/z* (positive ion mode) 427 [M+Na]^+^; HRESIMS (positive ion mode) *m/z* 427.1001 [M+Na]^+^ (calcd. C_20_H_20_NaO_9_ for 427.1005).

### 3.4. Anti-TMV Assays

The anti-TMV activities were tested using the half-leaf method [15]. Ningnanmycin (2% water solution), a commercial product for plant disease in China, was used as a positive control.

## 4. Conclusions

Three new xanthones **1**–**3** and five known analogues **4**–**8** were isolated from the acetone extract of the leaves of *Garcinia* *paucinervis*. All the compounds were evaluated for anti-tobacco mosaic virus (anti-TMV) activities. Compounds **1**–**3** showed potent anti-TMV activities, with inhibition rate, above 20%. Compound **1** had a lower IC_50_ value of 21.4 µM.

## Supplementary Materials

Supplementary materials (NMR spectra of **1**–**3**) can be accessed at: http://www.mdpi.com/1420-3049/18/8/9663/s1.
