# Supplementary material for: Three Novel Xanthones from Garcinia paucinervis and Their Anti-TMV Activity
Source: Molecules. 2013 Aug 13;18(8):9663–9. doi: 10.3390/molecules18089663 (PMC6269950; doi:10.3390/molecules18089663)

Supplementary Materials

Figure S1. ^{1}H-NMR spectrum of **1** (500 MHz, C_5D_5N).

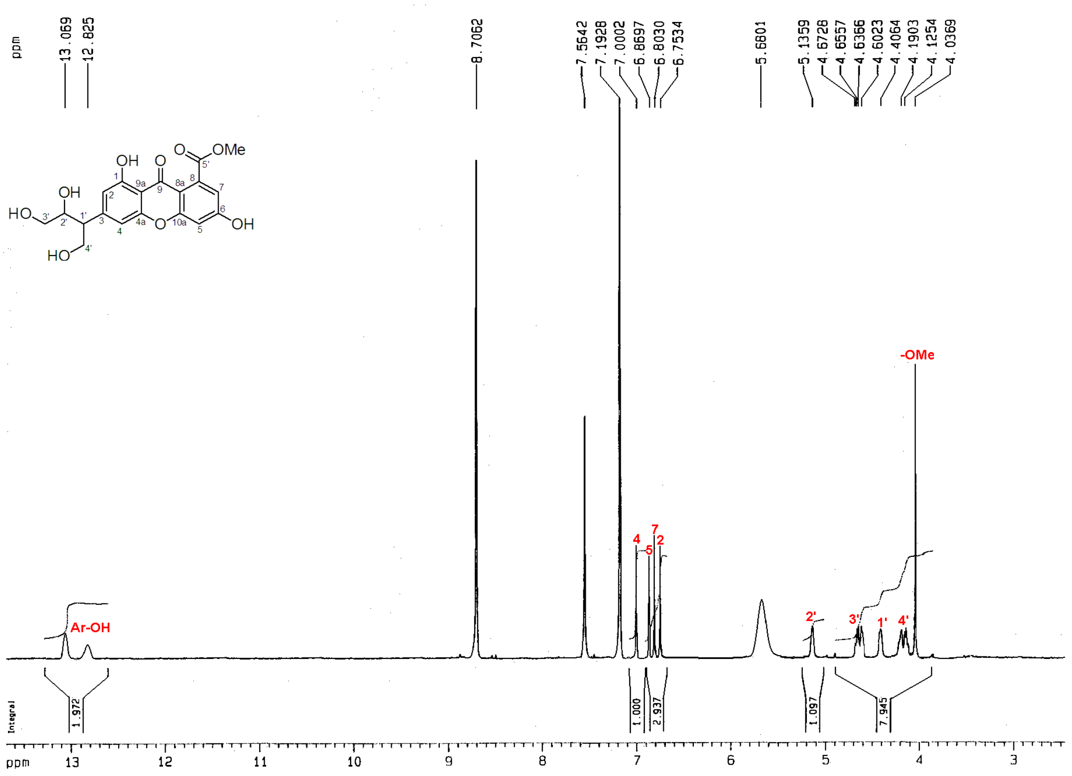

Figure S2. ^{13}C-NMR spectrum of **1** (125 MHz, C_5D_5N).

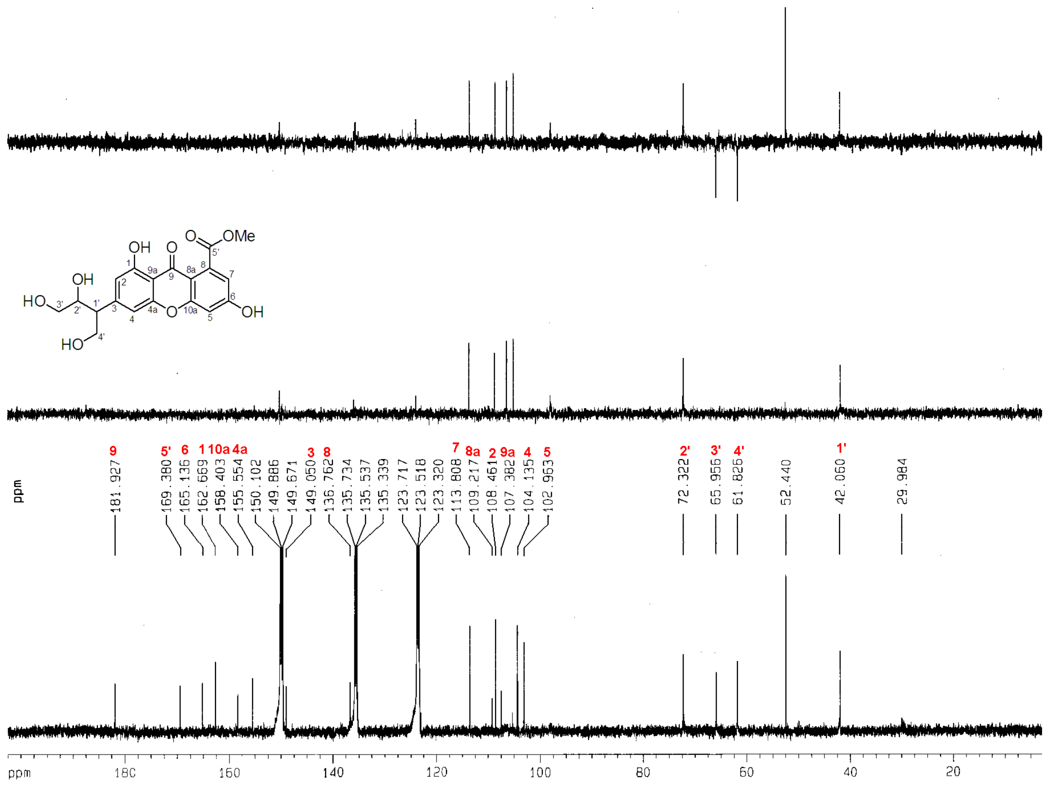

Figure S3. ^{1}H-NMR spectrum of **2** (500 MHz, C_5D_5N).

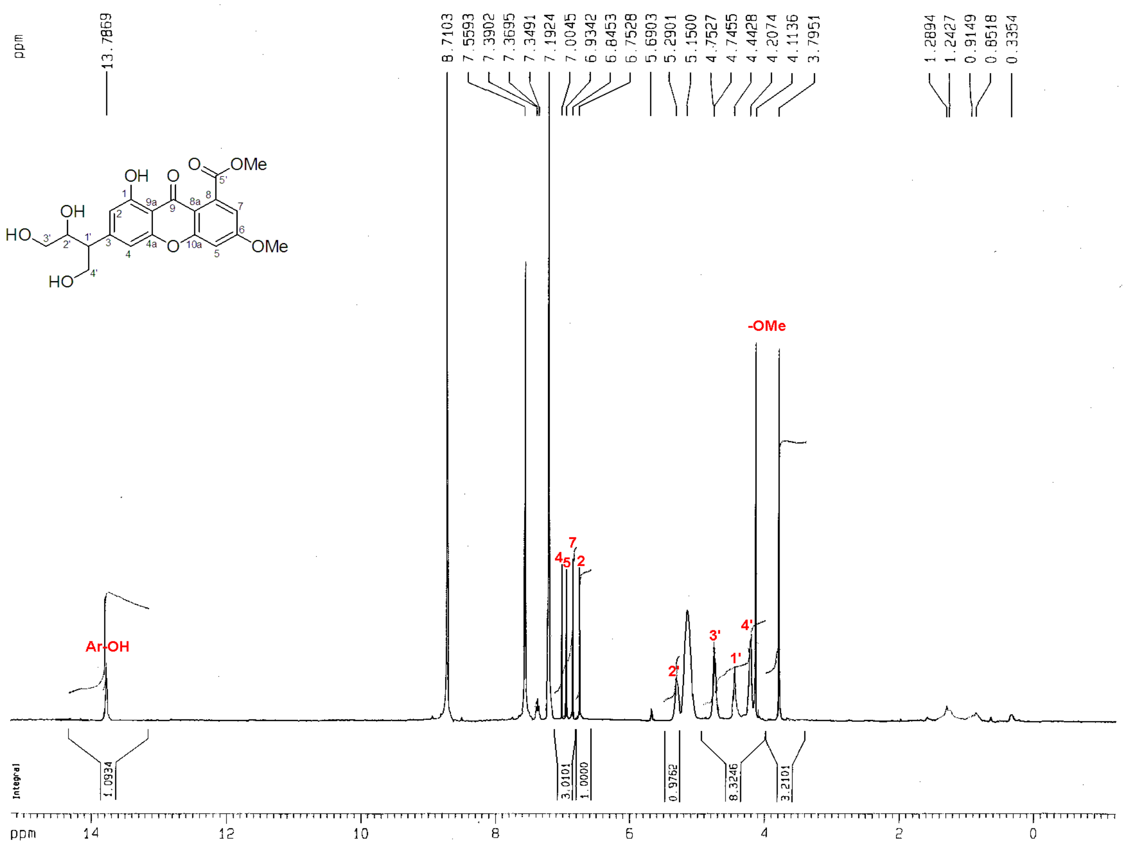

Figure S4. ^{13}C-NMR spectrum of **2** (125 MHz, C_5D_5N).

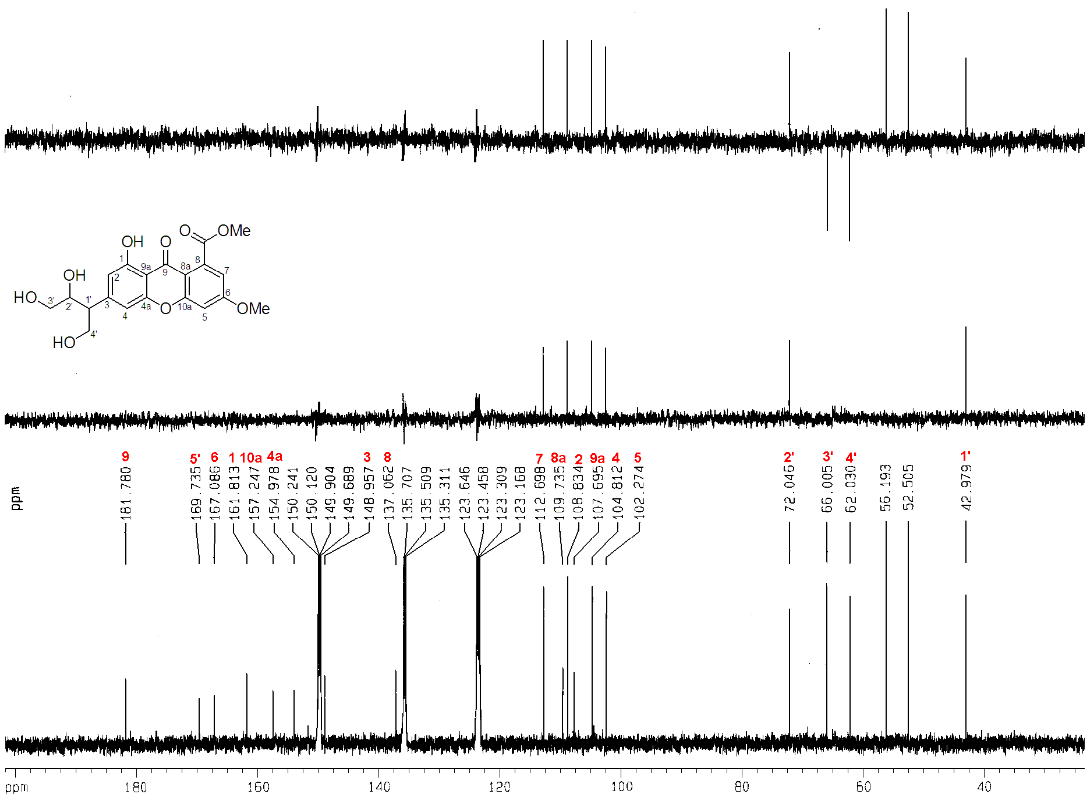

Figure S5. ^{1}H-NMR spectrum of **3** (500 MHz, C_5D_5N).

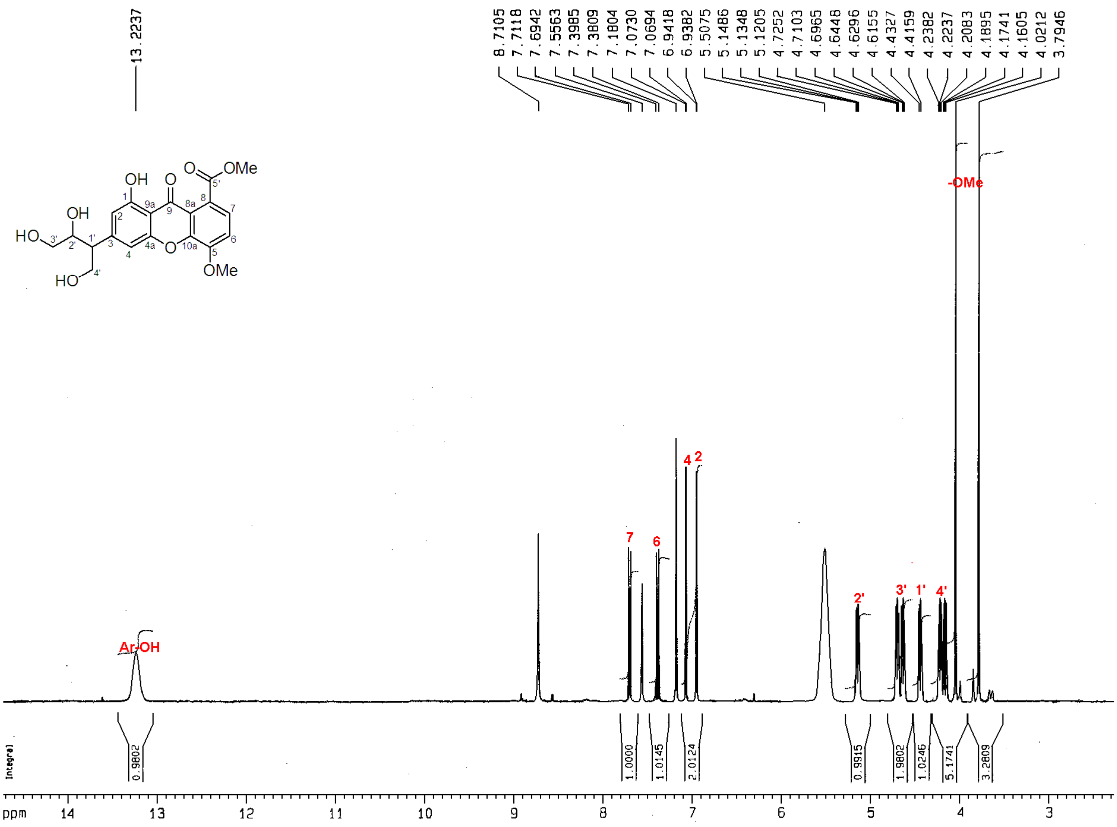

Figure S6. ^{13}C-NMR spectrum of **3** (125 MHz, C_5D_5N).

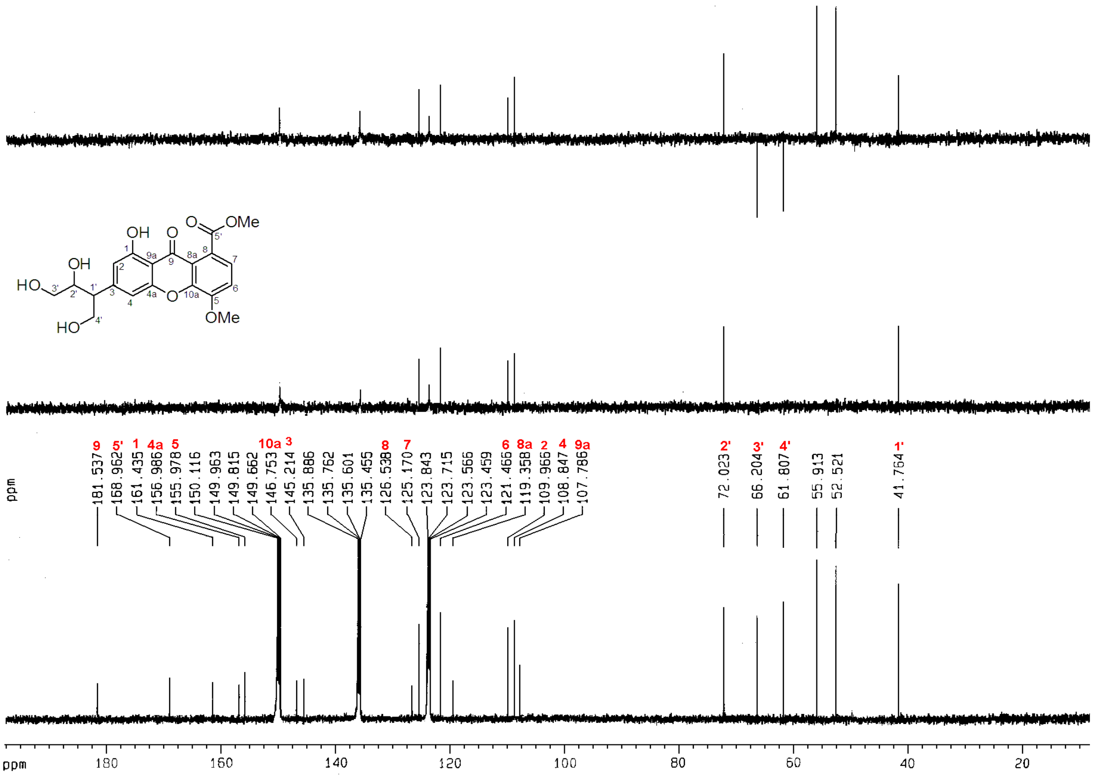

Supplement: Supplementary file 1 [file molecules-18-09663-s001.pdf]
